# Commissioning of Halcyon enhanced leaf model in the Eclipse treatment planning system: Focus on simple slit fields and VMAT dose calculation

**DOI:** 10.1002/acm2.70044

**Published:** 2025-02-17

**Authors:** Ryohei Miyasaka, Mari Shirai, Mitsunobu Igari, Yume Kojima, Yuki Kozawa, Toru Kawachi, Ryusuke Hara

**Affiliations:** ^1^ Division of Radiation Oncology Chiba Cancer Center Chiba Japan; ^2^ Medical Radiology Room Shimada General Medical Center Shimada Shizuoka Japan; ^3^ Department of Radiation Oncology Saitama Medical University International Medical Center Hidaka Saitama Japan; ^4^ Department of Radiology Imaging Sagamihara Kyodo Hospital Sagamihara Kanagawa Japan

**Keywords:** dose calculation, dose verification, dual‐layer MLC, ELM, VMAT

## Abstract

**Purpose:**

The dual‐layer multileaf collimator (MLC) in Halcyon adds complexities to the dose calculation process owing to the variability of dosimetric characteristics with leaf motion. Recently, an enhanced leaf model (ELM) was developed to refine the MLC model in the Eclipse treatment planning system. This study investigates the performance of the Halcyon ELM by verifying doses for simple slit fields and volumetric modulated arc therapy (VMAT) plans.

**Materials and methods:**

Dose calculations were performed with Acuros XB using the ELM. To commission the leaf–tip model, the dosimetric leaf gap (DLG) was calculated (referred to as DLG_ELM_) and compared with Halcyon measurements. The DLGs were assessed under conditions both with and without leaf trailing between the MLC layers. The tongue‐and‐groove (TG) model was evaluated by comparing leaf–edge profiles and the outputs of the asynchronous sweeping gap. Furthermore, eleven VMAT plans were validated against chamber doses and Delta4 measurements.

**Results:**

DLG_ELM_ demonstrated variation between layers, measuring 0.42 mm for the proximal layer and 0.23 mm for the distal layer, and showed a correspondence with the measured DLGs in 0.1 mm. Additionally, ELM reduced the discrepancy between calculated and measured DLGs when accounting for leaf trailing. In the TG model test, ELM calculations successfully mirrored the measured leaf–edge profiles. Moreover, the median dose difference between ELM calculations and chamber doses was −0.8% in asynchronous sweeping gaps. In the VMAT dose verification, the incorporation of ELM enhanced the target dose and resulted in a gamma pass rate (2%/2 mm) exceeding 95%.

**Conclusion:**

Halcyon ELM considerably improved the accuracy of simulating actual leaf–tip transmission, both with and without leaf trailing, and it effectively accounted for the additional blocking caused by TG design. Furthermore, the introduction of ELM in Eclipse considerably enhanced the VMAT dose calculation. ELM addresses the limitations of traditional leaf models and reduces uncertainties in Halcyon dose calculations.

## 1 INTRODUCTION

Halcyon (Varian Medical Systems, Palo Alto, CA, USA) is an O‐ring linear accelerator (linac) with a single 6 MV flattening filter‐free beam, a rapid gantry rotation speed of four rotations per minute, and a high dose rate of up to 8 Gy/min.^1^ Furthermore, it incorporates a dual‐layer multileaf collimator (MLC) with stacked and staggered leaves in the field aperture. This dual‐layer MLC effectively reduces leakage dose by blocking inter‐leaf gaps.^2^ These mechanical features allow Halcyon‐based high‐precision radiotherapy to achieve a quality level that is consistently equivalent to or better than that of other established advanced linacs.^3^, ^4^, ^5^, ^6^ Conversely, the dual‐layer MLC introduces additional complexities in dose delivery because the transmission around the rounded leaf tip varies based on the leaf‐trailing pattern used for both layers. When the leaf‐trailing distance is larger, the leaf–tip transmission is comparable to that of a standard MLC with a single layer. However, as the trailing distance decreases, the leakage dose approaches that of a monoblock collimator. Hernandez et al. referred to this variation in transmission as the “leaf‐trailing effect”.^7^

Previous studies have indicated that the accuracy of dose calculations in high‐precision radiotherapy techniques, such as intensity‐modulated radiation therapy (IMRT) and volumetric modulated arc therapy (VMAT), is dependent on the quality of the MLC model in the treatment planning system (TPS).^8^, ^9^, ^10^, ^11^, ^12^ The conventional leaf model (CLM) used in the Eclipse TPS (Varian Medical Systems, Palo Alto, CA, USA) replicates MLC characteristics—including dose leakage from the leaves and additional shielding from the tongue‐and‐groove (TG) design—using just three parameters: MLC transmission, dosimetric leaf gap (DLG), and TG width.^13^ MLC transmission is defined as the average of intra‐ and inter‐leaf transmission and is considered constant over the entire leaf structure. The DLG parameter simulates the increase in field size owing to leaf–tip transmission, thereby reducing uncertainties in dose calculations that arise from straightforward MLC models with straight leaf ends. TG width reproduces an exposed tongue in the treatment field and adjusts the delivered fluence during the dose calculation by blocking a portion of the radiation beam. While these parameters effectively and simply capture MLC characteristics, various reports have highlighted systematic uncertainties in CLM for advanced treatment planning, such as VMAT.^7^, ^10^, ^14^, ^15^, ^16^ Hernandez et al. found that the Halcyon CLM lacked an explicit model for the leaf tip and was unable to replicate the leaf‐trailing effect.^7^ They suggested introducing new challenges for the Eclipse TPS, such as the necessity for separate parameters for each MLC layer and addressing the trailing effect. In the VMAT dose verifications for Halcyon, Lim et al. reported a dose discrepancy of −1.28% ± 0.80% (mean ± SD) for the AAA (version 15.6, Varian Medical Systems, Palo Alto, CA, USA) across 10 patient treatment plans.^14^ Miyasaka et al. found that the doses calculated using Acuros XB (version 15.6, Varian Medical Systems, Palo Alto, CA, USA) were underestimated in 32 VMAT dose verifications, with an average dose difference of approximately −1.3%, and the gamma index pass rate with the 2%/2 mm criterion fell short of 95%.^15^ They recommended that modifying the leaf–tip model could effectively reduce dose calculation uncertainties and was essential for the commissioning of the Halcyon MLC model. Similarly, Pérez Azorín et al. examined the failures in dose verifications of sliding window IMRT plans, performing a comprehensive analysis that linked discrepancies to limitations in the MLC model and particular characteristics of leaf sequences.^16^ They concluded that inadequate modeling of the leaf tip and very low leaf speeds contribute to uncertainties in dynamic MLC dose calculations.

Recently, an enhanced leaf model (ELM) was developed for the MLC model in the Eclipse TPS. This model was created through ray tracing of the actual leaf design, incorporating features such as the rounded tip shape of the MLC leaves, the drive screw cutout, and the thickness of the leaf body.^13^ The ELM is designed for three MLC types manufactured by Varian Medical Systems, namely, High‐definition 120, Millennium 120 and Halcyon SX, and can be used for the dose calculations with AAA, Acuros XB and Photon Optimizer Algorithm (PO, Varian Medical Systems, Palo Alto, CA, USA) in Eclipse version 18.0 and later. When the ELM is used in the dose calculation process, the uncertainty arising from a simple MLC model can be reduced because it takes into account the change of photon fluence due to the path length inside the leaf body and the effect of the rounded leaf–tip on the penumbra shape. Therefore, the ELM is expected to more accurately replicate the characteristics of dual‐layer MLC and enhance high‐precision dose calculations in Halcyon. Van Esch et al. have recently reported that the Halcyon ELM considerably improves dose calculation accuracy when the leaf tips of both MLC layers are aligned during either static or dynamic MLC sequences.^17^ In the static case, the ELM agreed with film or array detector measurements within 4% in narrow dose profiles, which was a notable improvement compared to the more than 30% deviation observed for the CLM. In addition, the CLM overestimated the integral doses of the dynamic leaf sequences by 7%–10% relative to the measurements, whereas the ELM reduced these deviations to less than 2% regardless of off‐axis position. On the other hand, their study did not assess the accuracy of the MLC model at each layer or explore the potential for calculating the trailing effect. Additionally, they did not address enhancements to dose calculations in high‐precision treatment plans. In this study, we evaluate the performance of the Halcyon ELM in accurately representing the MLC characteristics of each layer and the leaf‐trailing effect through dose verification for single or dual‐layer MLC slit fields. Furthermore, we assess the dosimetric benefits of incorporating ELM into the dose calculation process through dose verifications for VMAT plans.

## 2 MATERIALS AND METHODS

In this study, all treatment plans were developed in the Eclipse research environment (version 18.0) and calculated using the Acuros XB dose calculation algorithm. The simple slit field plans were exported from the TPS in Digital Imaging and Communications in Medicine (DICOM) format and subsequently modified with an in‐house program (MATLAB R2021a, Mathworks, Natick, MA, USA) owing to the inability to manually assign leaf motion in Eclipse TPS. In each layer, all leaf positions were defined in the isocenter plane. The VMAT plans were optimized using the PO (version 18.0). For dose calculations, a grid size of 1 mm was established for the simple slit field plans, while a 2 mm grid size was used for the VMAT plans.

### 2.1 Evaluation of the leaf–tip model

To quantify the leaf–tip transmission, the DLG was assessed in the proximal single‐layer, distal single‐layer, and dual‐layer MLC sequences. To determine the DLG at the beam axis, the dose ratio between the sweeping gap field and a reference field of 10 cm × 10 cm (*D*_ref_) was measured using an ionization chamber (30013, PTW, Freiburg, Germany) positioned at a depth of 10 cm and a source‐to‐chamber distance of 100 cm. The gap widths were varied from 2 to 20 mm in 2 mm increments in a 10 cm × 10 cm square field. The sweeping distance was consistently maintained at 12 cm across all sequences, while the leaf speed was set at 5 mm/s between all control points. The outputs of the sweeping gap field were adjusted by subtracting the contribution of inter‐ and intra‐leaf transmission for each layer. This leaf transmission was determined from the chamber readings in the static field fully blocked by the proximal or distal single‐layer MLCs. The DLG was calculated from the negative intercept of the linear fits between the dose ratio and sweeping gap width, measured in three Halcyon linacs (version 3.0) from different institutions for both single‐layer and dual‐layer MLC sequences.

**FIGURE 1:**
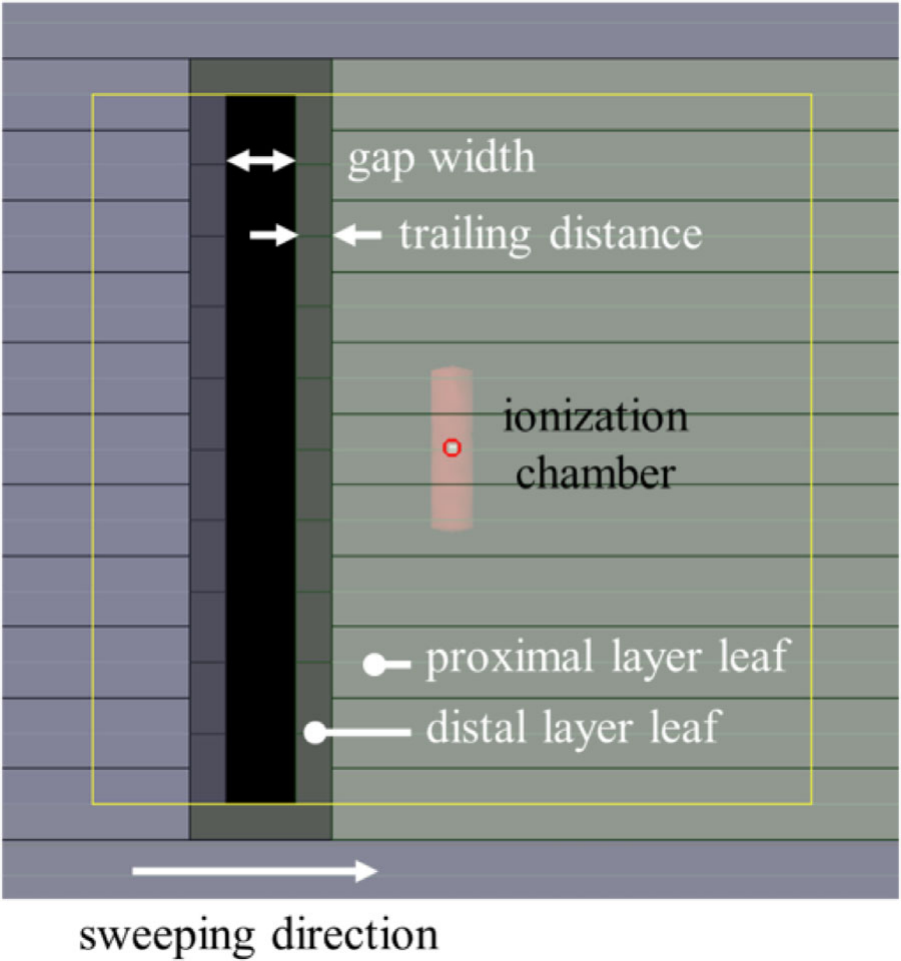
Beam's eye view of the sweeping gap field controlled by the dual‐layer sequences. One leaf layer is trailed at a fixed distance by another leaf layer. The trailing distance was defined as the distance between the leaf tips in the dual‐layer multileaf collimator (MLC).

Furthermore, the leaf‐trailing effect was assessed using the “trailing sweeping gap test”.^7^ As illustrated in Figure 1, the sweeping gap field was trailed by an additional layer at a fixed distance. In the previous reports, the distance between the leaf tips was named the “trailing distance”.^7^ The DLG was determined using various trailing distances (*t*) of 0, 0.5, 1, 2, 3, 4, 5, 10, or 20 mm. Corrections for inter‐ and intra‐leaf transmission were made based on the field size shielded by only a single layer because the transmission through both layers was less than 0.01%. The trailing sweeping gap test was performed only on Halcyon #A.

The sweeping gap fields and reference field were simulated using Eclipse TPS and calculated with CLM or ELM in a water phantom. For each single‐layer and dual‐layer MLC, the DLGs for CLM (referred to as DLG_CLM_) and ELM (referred to as DLG_ELM_) were determined and compared to experimental measurements.

### 2.2 Test of the TG model

**FIGURE 2:**
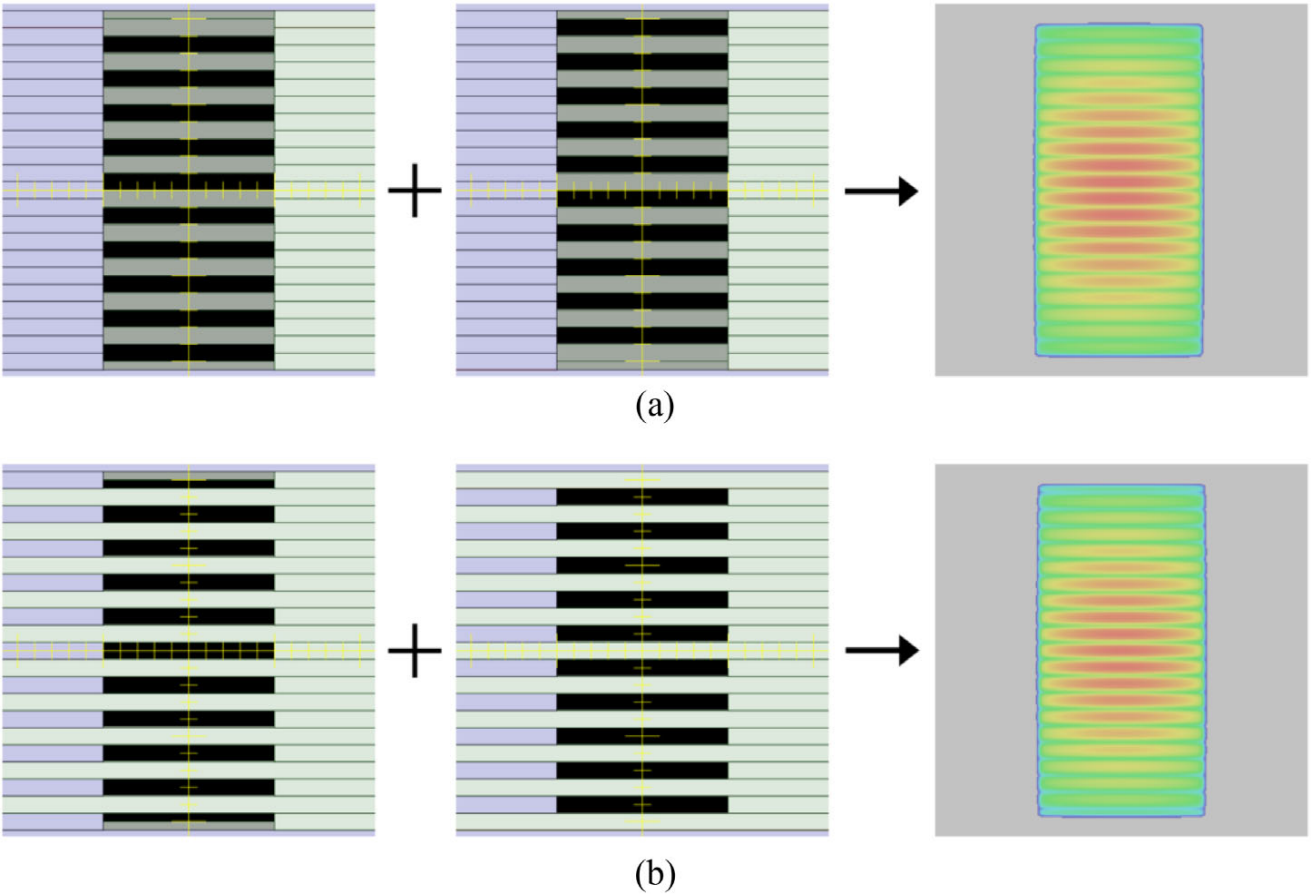
Beam's eye view of a combination of two static slit‐fields and calculated dose distributions using (a) the proximal layer multileaf collimator (MLC) and (b) the distal layer MLC.

As illustrated in Figure 2, two static MLC slit fields were combined to assess the TG effect in the (a) proximal and (b) distal layer. The first static slit field, measuring 10 cm × 20 cm, was configured to open the MLCs at even‐numbered positions while keeping their neighboring leaves closed. Conversely, the second field was designed to close the even leaves and open the odd ones. The dose profiles of these static slit fields were measured using a radiochromic film (Gafchromic EBT3, Ashland Inc., USA) placed in a water‐equivalent solid phantom (Toughwater, Kyoto Kagaku Co., Japan) at a depth of 10 cm. To assess the TG model, the combination of two static slit fields was calculated using CLM or ELM. Subsequently, the calculated dose profiles were validated against film measurements using a one‐dimensional global gamma index, applying a 3% dose difference criterion and a 2‐mm distance‐to‐agreement (DTA) criterion, with a lower dose threshold of 10% (3%/2 mm, 10%). The absolute dose differences between each measured and calculated dose point pair were normalized based on the maximum measured dose, and the pass rate (3%/2 mm, 10%) was evaluated.

**FIGURE 3:**
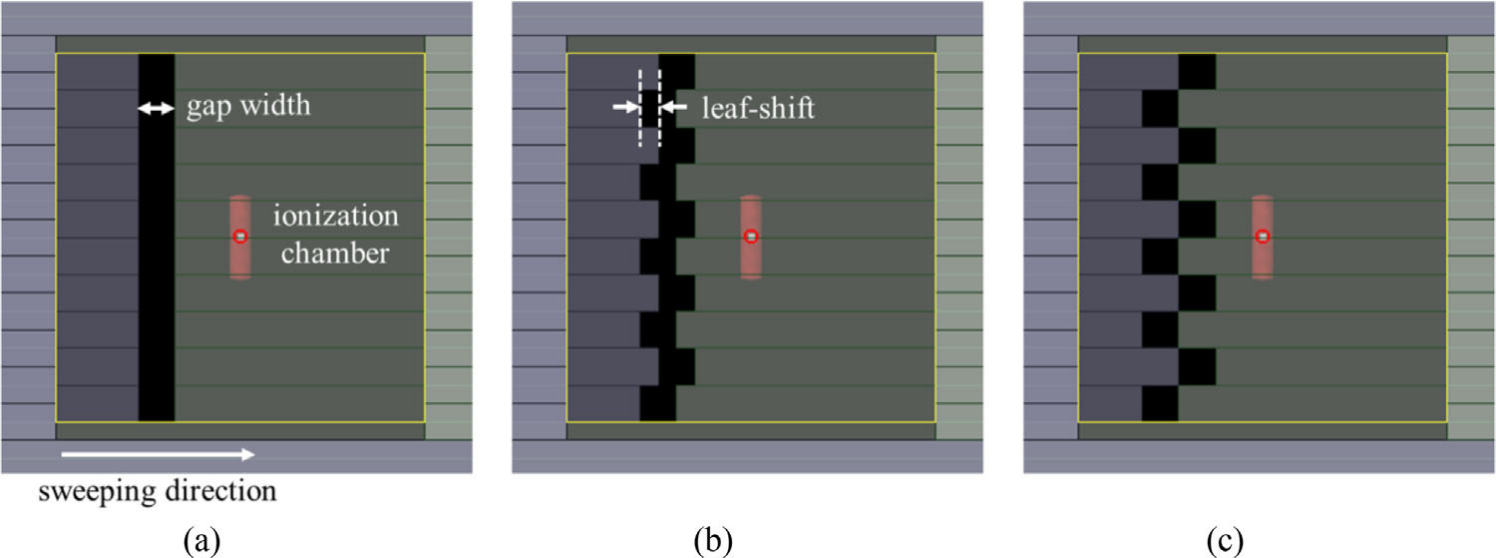
Beam's eye view of the asynchronous sweeping gap test based on the tongue‐and‐groove (TG) fraction of (a) 0, (b) 0.5, and (c) 1.0. A TG fraction of 0 indicates uniformly extended gaps, while a higher TG fraction indicates a more pronounced TG effect.

To ensure quality assurance (QA) of the TG model in dynamic MLC cases, we performed an “asynchronous sweeping gap test” for each layer.^18^ Traditional sweeping gap tests typically feature uniformly extended leaves, which do not demonstrate any TG effect. In contrast, the asynchronous sweeping gap test accounts for the TG effect in the irradiation field by adjusting the positions of neighboring leaves. The ratio of leaf shift (*s*) to gap width (*g*) is defined as the TG fraction (*s*/*g*); a TG fraction of 0 indicates uniformly extended gaps, while a higher TG fraction indicates a more pronounced TG effect, as illustrated in Figure 3. The sweeping gaps of 5, 10, and 20 mm were analyzed using various TG fractions of 0, 0.5, and 1.0, respectively. Dose measurements were performed with an ionization chamber (30013) located at the isocenter and adjusted to account for daily variations in the linac output. Additionally, calculated doses (*D*_calc_) from CLM or ELM in the sweeping gap fields were compared to the chamber measurements (*D*_meas_) using the formula “(*D*_calc_ − *D*_meas_)/*D*_meas_ × 100”.

### 2.3 Dose verification of the VMAT treatment plan

The dosimetric benefits of incorporating ELM were assessed through dose verifications of VMAT treatment plans. As detailed in Table 1, VMAT plans were generated using three arcs for both benchmark and nonclinical patient cases.^19^, ^20^ These plans were then recalibrated using either CLM or ELM in virtual phantoms that simulated the QA devices: a water‐equivalent cubic phantom (Toughwater) with an inserted ionization chamber (31021, PTW, Freiburg, Germany) and the commercial biplanar diode array device (Delta4 Phantom+ with Plastic Water DT, ScandiDos Inc., Ashland, VA, USA). The VMAT plans were delivered through the Halcyon linac to each QA device. Before all measurements, the dose per monitor unit was obtained in accordance with the standard dosimetry protocol, and the daily linac output deviation was corrected.

**TABLE 1:** The summary of plan characteristics for each volumetric modulated arc therapy (VMAT). Cases #1 to #3 are benchmark,^19^ and cases #4 to #11 are treatment plans for non‐clinical patients.^20^

Case #	Target	Prescribed dose	Target volume (cc)	Treatment strategi
1	Mock prostate cancer	1.8 Gy × 42 fraction	81.0	Standard VMAT
2	Mock head and neck cancer	2.0 Gy × 25 fraction	509.4	Standard VMAT
3	C‐shape	2.0 Gy × 25 fraction	166.3	Stereotactic VMAT
4	Prostate cancer	3.0 Gy × 20 fraction	127.7	Standard VMAT
5	Head and neck cancer	2.0, 1.8 or 1.6 Gy × 35 fraction	1297.3	Simultaneous integrated boost
6	Head and neck cancer	2.0, 1.8 or 1.6 Gy × 35 fraction	596.6	Simultaneous integrated boost
7	Esophagus cancer	1.8 Gy × 28 fraction	381.0	Standard VMAT
8	Lung metastases	10.0 Gy × 5 fraction	11.3	Stereotactic VMAT
9	Spine metastases	7.0 Gy × 5 fraction	38.2	Stereotactic VMAT
10	Pancreas cancer	2.0 Gy × 26 fraction	69.4	Standard VMAT
11	Liver metastases	10.0 Gy × 5 fraction	65.6	Stereotactic VMAT

In the single‐point dose verifications, the calculated doses were compared to the chamber doses, with relative dose differences assessed using the formula “(*D*_calc_ − *D*_meas_)/*D*_meas_ × 100”. Additionally, the calculated dose profiles were verified against Delta4 measurements in absolute dose mode, employing a three‐dimensional global gamma index with a 2% dose difference criterion and a 2‐mm DTA criterion, applying a lower dose threshold of 10% (2%/2 mm, 10%). In this analysis, the absolute dose difference between each pair of measured and calculated dose points was normalized using the maximum measured dose value (global normalization). This method of global normalization is recommended by international guidelines for IMRT measurement‐based dose verification.^21^ Additionally, the gamma passing rate (2%/2 mm, 10%) was assessed for this analysis.

## 3 RESULTS

### 3.1 Evaluation of the leaf–tip model

Table 2 presents the measured DLGs for three Halcyon linacs, including DLG_CLM_ and DLG_ELM_ for the proximal single‐layer, distal single‐layer, and dual‐layer MLC sequences. The measured DLGs among the linacs demonstrated consistency in 0.02 mm for the proximal single‐layer, 0.07 mm for the distal single‐layer, and 0.03 mm for the dual‐layer. DLG_CLM_ was approximately 0.04 mm for each MLC layer, while DLG_ELM_ showed variability between layers, measuring 0.42 mm for the proximal layer and 0.23 mm for the distal layer. Additionally, DLG_ELM_ values exhibited agreement with the measured DLGs, all in 0.1 mm.

**TABLE 2:** Dosimetric leaf gaps (DLG) measured in three Halcyon linacs, and DLG was calculated with conventional leaf model (CLM) and enhanced leaf model (ELM). DLGs were determined using each single‐layer and dual‐layer sweeping gap MLC fields.

	Measured DLG (mm)			Calculated DLG (mm)	
Leaf‐sequence	Machine A	Machine B	Machine C	CLM	ELM
Proximal single‐layer	0.434	0.440	0.421	0.043	0.421
Distal single‐layer	0.314	0.261	0.252	0.050	0.228
Dual‐layer	−0.465	−0.492	−0.490	0.030	−0.365

**FIGURE 4:**
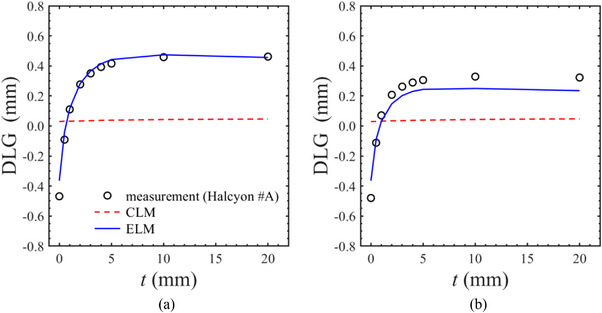
The change of the dosimetric leaf gap (DLG) as a function of the leaf trailing distance (*t*) for the (a) proximal and (b) distal layers. No change in DLG was observed owing to leaf trailing in the conventional leaf model (CLM), while the calculated DLG based on the enhanced leaf model (ELM) offered a more accurate characterization of the dual‐layer multileaf collimator (MLC).

In the trailing sweep gap test, the measured DLGs were similar to those of the single layer at trailing distances ranging from 5 to 20 mm; however, they decreased sharply at distances less than 5 mm, as illustrated in Figure 4. In CLM dose calculation, no change in DLG was observed owing to leaf trailing. Conversely, Halcyon ELM offered a more accurate characterization of the dual‐layer MLC, reducing the discrepancy between the calculated and measured DLGs for dual‐layer leaf motion with leaf trailing.

### 3.2 Test of the TG model

**FIGURE 5:**
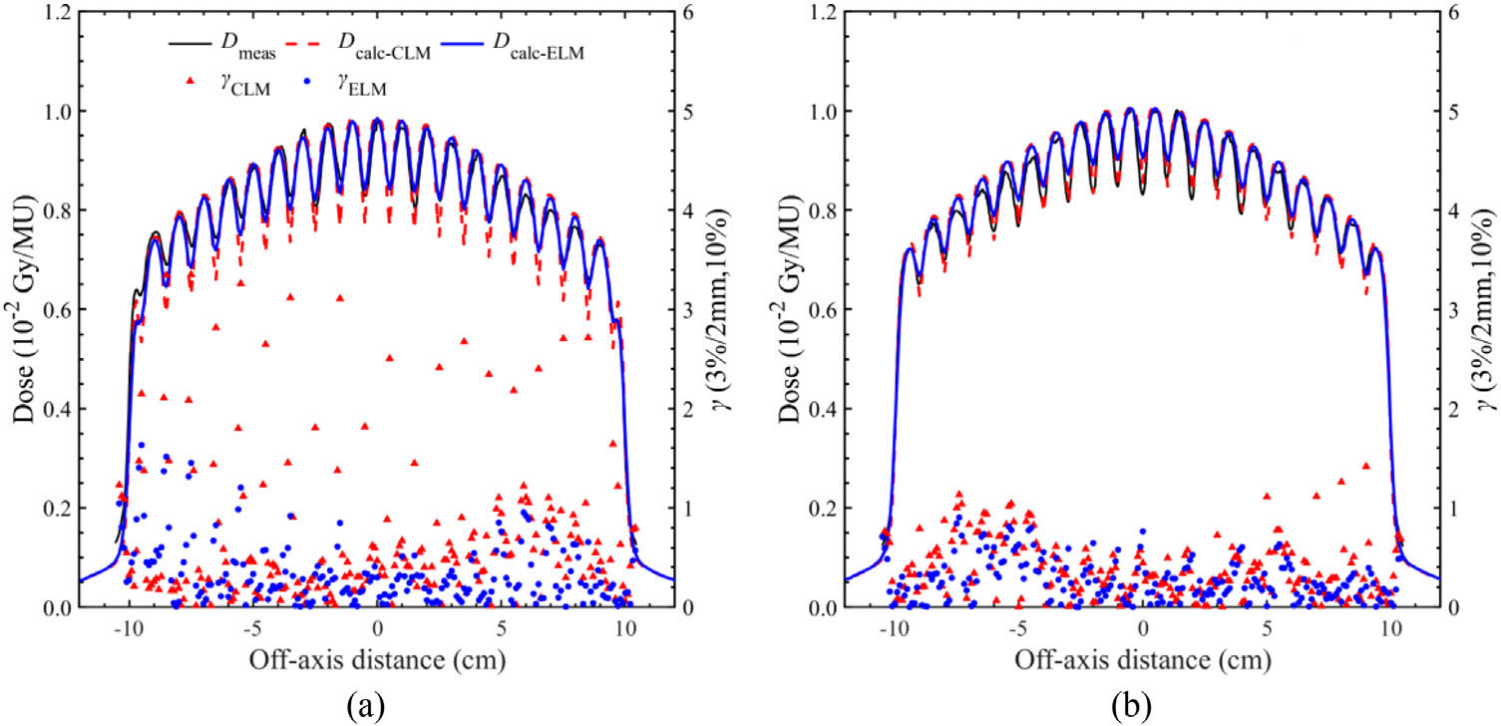
The measured and calculated dose profiles regarding the combination of two static slit‐fields and a one‐dimensional global gamma index, applying a 3% dose difference criterion and a 2‐mm distance‐to‐agreement (DTA) criterion, with a lower dose threshold of 10% (3%/2 mm, 10%). (a) In the proximal layer, the tongue‐and‐groove (TG) effect in the conventional leaf model‐based dose calculation (*D*_calc‐CLM_) underestimated the measured dose (*D*_meas_), while the calculated dose based on the enhanced leaf model (*D*_calc‐ELM_) was reproduced the film measurement. (b) The TG effect on the distal layer showed good agreement with the measurements in each leaf model. Near the central axis, the *D*_calc‐ELM_ tended to be slightly higher than the *D*_meas_.

Figure 5 shows the measured and calculated dose profiles for the combination of two static slit fields, while Table 3 presents a summary of the gamma pass rate (3%/2 mm, 10%) for both the CLM and ELM dose calculations. In the proximal layer, ELM calculations (*D*_calc‐ELM_) closely matched the measured dose profile, achieving gamma pass rates exceeding 96% (3%/2 mm, 10%). In contrast, the dose profiles generated using the CLM (*D*_calc‐CLM_) demonstrated lower accuracy in capturing the TG effect. On the other hand, the TG effect on the distal layer showed good agreement with measurements in each leaf model. Near the central axis, the *D*_calc‐ELM_ tended to be slightly higher than the measured dose profile.

**TABLE 3:** The summary of the pass rate of gamma index with the criterion of 3%/2 mm between the dose calculation with conventional leaf model (CLM) or enhanced leaf model (ELM) and film measurements regarding the tongue‐and‐groove effect in static slit‐fields.

	Gamma pass rate (%)	
	CLM	ELM
Proximal layer MLC	79.4	96.2
Distal layer MLC	95.7	100

**FIGURE 6:**
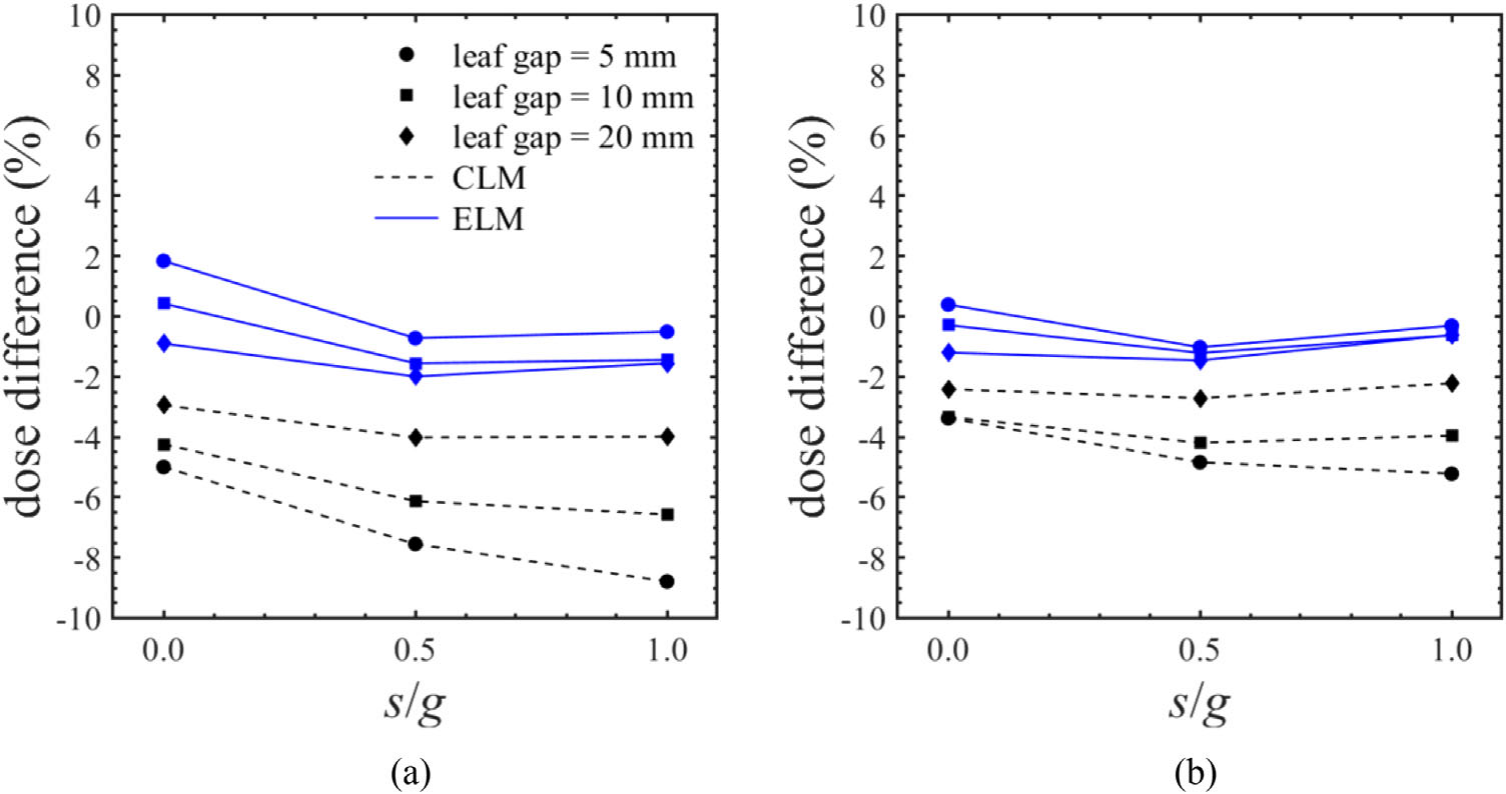
The dose difference between the conventional leaf model (CLM) or enhanced leaf model (ELM) dose calculations and the chamber measurements in the asynchronous sweeping leaf gap of 5, 10, and 20 mm using the different tongue‐and‐groove fraction (*s*/*g*). In both (a) proximal layer and (b) distal layer, the CLM underestimated the actual linac output, whereas ELM improved its uncertainty.

In the dynamic MLC cases, Figure 6 illustrates the dose differences between the CLM and ELM dose calculations compared to chamber measurements across asynchronous sweeping gaps of 5, 10, and 20 mm using various TG fractions. The median dose differences were −4.1% (range: −8.8% to −2.2%) for CLM and −0.8% (range: −1.9% to 1.8%) for ELM.

### 3.3 Dose verification of the VMAT treatment plan

**FIGURE 7:**
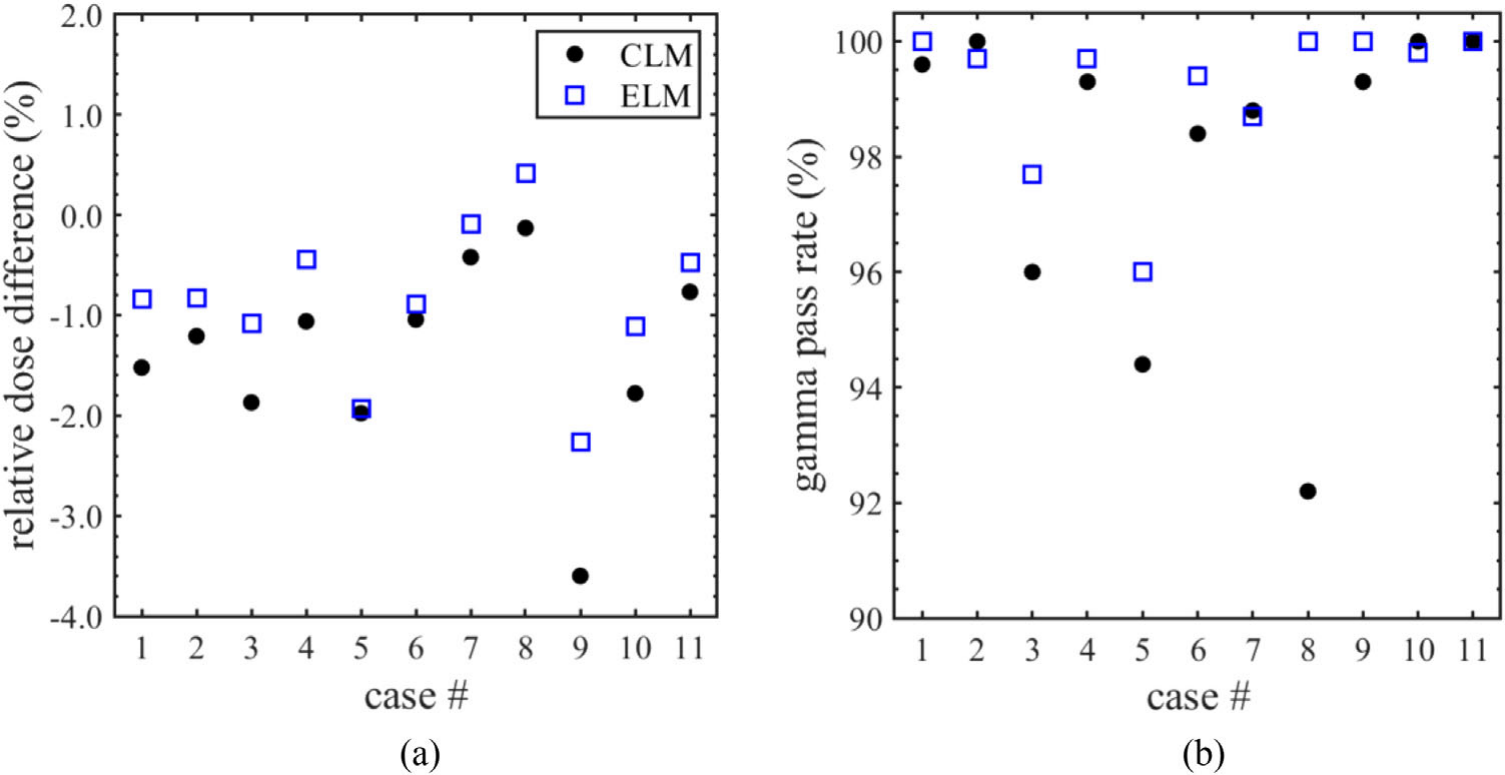
The summary of (a) relative dose difference and (b) gamma pass rate with the criterion of 2%/2 mm calculated using conventional leaf model (CLM) and enhanced leaf model (ELM) in each volumetric modulated arc therapy (VMAT) plan.

Figure 7 shows (a) the relative dose difference and (b) the gamma pass rate (2%/2 mm, 10%) for each VMAT treatment plan. In the single‐point dose verifications, CLM dose calculations were found to underestimate the chamber measurements, resulting in a mean dose difference of −1.40% (with a range of −3.59% to −0.13%). In contrast, ELM calculations yielded higher doses, improving the mean dose difference to −0.87% (with a range of −2.26% to 0.41%). The gamma pass rate for CLM had a mean of 98.0% (ranging from 92.2% to 100%) but fell below 95% for the C‐shape (case #3), head and neck (case #5), and small lung cancer (case #8) treatment plans. In the dose verification for ELM, the mean gamma pass rate reached 99.2%, with a range of 96.0%–100%. The calculated dose distributions closely matched the Delta4 measurements, achieving an acceptable level in all plans. These findings suggest that ELM effectively reduced the systematic discrepancies between the measured and calculated doses in VMAT treatment plans.

## 4 DISCUSSION

To assess the accuracy of dose calculations using the ELM in the Eclipse TPS, we performed verifications comparing dose calculations with measurements across simple slit fields and VMAT dose deliveries. During the commissioning of the leaf–tip model, DLGs were evaluated using both single‐layer and dual‐layer MLC sequences. The measured DLGs from three Halcyon linacs demonstrated good agreement. A previous study found that the mean DLG for five Halcyon linacs was 0.44 mm for the proximal single‐layer (ranging from 0.36 to 0.48 mm), 0.28 mm for the distal single‐layer (ranging from 0.12 to 0.38 mm), and −0.49 mm for the dual‐layer (ranging from −0.42 to −0.62 mm).^7^ The measured DLGs closely aligned with previous studies, indicating that Halcyon leaf–tip transmission maintained comparable levels worldwide. Whether using a single‐layer or dual‐layer MLC sequence, DLG_CLM_ remained unchanged, while DLG_ELM_ reproduced the measured DLG with a discrepancy of less than 0.1 mm. Under leaf‐trailing conditions, the measured DLG sharply declined when the trailing distance was under 5 mm, and furthermore, the DLG showed negative values ​​when the trailing distance approached zero. Previous study discussed that the negative DLG was due to the occlusion of the photon source: when both MLC layers define a similar gap size, the combined source occlusion by each layer reduces the photon fluence passing through a narrow leaf gap and could contribute to a negative DLG.^7^, ^17^ It has been previously observed that CML does not account for this dose reduction.^7^, ^15^ ELM effectively addressed this challenge by implementing an accurate leaf–tip model. Note that the DLG parameter in CLM for the Halcyon is set to 0.1 mm in advance and unified in all institutes. In this study, the DLG_CLM_ showed approximately 0.04 mm ± 0.01 mm for all MLC sequences, which was slightly smaller than the preset DLG parameter of 0.1 mm. Hernandez et al. reported that the calculated DLG using Halcyon CLM was 0.13 mm ± 0.01 mm regardless of the MLC layer, of the trailing distance. Their DLG values were slightly larger than the fixed 0.10 mm parameter preconfigured in the CLM.^7^ The TPS dose calculation may have uncertainties even though the calculation grid size and dynamic MLC motion. These uncertainties may directly affect the determination of DLG and will be limitations in this study.

In the TG model test, the ELM provided a more accurate reproduction of the additional blocking with the tongue and was better aligned with the asynchronous sweeping gap output. Notably, it was observed that the CLM demonstrated lower accuracy in simulating the TG effect in the proximal layer. Previous studies have indicated that the basic TG model can compromise dose calculation accuracy in advanced treatment plans such as IMRT and VMAT.^10^, ^18^ Vieillevigne et al. reported that CLM dose calculations for VMAT plans were underestimated relative to the measurements, attributed to insufficient modeling of the TG effect in the Eclipse TPS.^10^ The TG effect is modeled in the dose calculation algorithms by extending the leaf projections in the direction perpendicular to the leaf motion. This parameter is not user‐configurable in the Eclipse, and it depends on the MLC model or dose calculation algorithm version. According to the latest vender documentation, the ELM takes into account the change of transmission due to path length inside the leaf body and the dosimetric effect of the rounded leaf–tip on the penumbra shape.^13^ Although it is not clear whether current ELM models the more detailed TG design, the uncertainties in dose calculations will be reduced by employing the ELM, as illustrated in Figures 5 and 6 and Table 3. Of note, for the static case of the distal MLC layer, the ELM calculation slightly underestimated the TG effect near the central axis. This result suggests that more accurate TG models will be needed in the future.

In the VMAT dose verification, the implementation of ELM increased the target dose by an average of 0.54% and resulted in a gamma pass rate (2%/2 mm, 10%) exceeding 95% across all cases, as illustrated in Figure 6. Our previous study suggested that to enhance the Halcyon CLM in the Eclipse TPS, the DLG parameter was specifically adjusted for VMAT dose calculations.^15^ While tuning the DLG is an effective strategy for compensating for CLM, it does not adequately replicate the leaf‐trailing effect or the TG effect. In contrast, ELM successfully addressed this limitation and enhanced dose calculations in the Halcyon system. In prior reports assessing the dose calculation accuracy for Halcyon VMAT treatment plans, several authors compared the differences between the AAA (version 15.6) dose calculations and actual measurements. Lim et al. found a dose discrepancy of −1.28% ± 0.80% (mean ± SD) when comparing AAA results to chamber measurements across 10 VMAT dose verifications.^14^ Similarly, Tamura et al. reported gamma pass rates of 97.82% ± 2.61% for prostate cancer and 96.27% ± 2.13% for head and neck cases, using a criterion of 2%/2 mm and 10%.^22^ The results obtained in this study align closely with those of the CLM owing to both AAA and Acuros XB in Eclipse employing the same MLC model. This indicates that the ELM in Halcyon contributes similarly to improving dose calculation accuracy for AAA. However, previous studies have not assessed the accuracy of the comparison of dose calculation between Acuros XB and AAA for Halcyon. Consequently, we plan to investigate AAA dose calculations using ELM in our future research.

## 5 CONCLUSIONS

In this study, we evaluated the Halcyon ELM's performance in accurately reproducing the MLC characteristics of each layer through dose verification for simple slit fields. The Halcyon ELM demonstrated a greater ability to simulate the actual leaf–tip transmission of dual‐layer MLC, both with and without leaf trailing, while also effectively accounting for the additional blocking owing to the TG design. Additionally, the verification results for VMAT plans using dual‐layer MLC showed considerable improvement when the ELM was incorporated into the dose calculation process. By addressing the limitations of CLM and reducing uncertainties in Eclipse dose calculations, the Halcyon ELM offers a dosimetric benefit that can enhance efficiency in clinical practice.

## AUTHOR CONTRIBUTIONS

Ryohei Miyasaka conceived of the presented idea. All authors conceived and planned the work that led to the paper. Mari Shirai, Mitsunobu Igari, Yume Kojima, and Yuki Kozawa performed the experiment. Ryohei Miyasaka and Toru Kawachi discussed the uncertainty of the Halcyon enhanced leaf model. Ryusuke Hara supervised the study. Ryohei Miyasaka analyzed the data, and all authors discussed the analysis method and results. All authors wrote the paper and approved the final version.

## CONFLICT OF INTEREST STATEMENT

The authors are involved in a collaboration with Varian Medical Systems and financial support was provided.
